# Development and Evaluation of Clove and Cinnamon Supercritical Fluid Extracts-Loaded Emulgel for Antifungal Activity in Denture Stomatitis

**DOI:** 10.3390/gels8010033

**Published:** 2022-01-04

**Authors:** Meenakshi Srinivas Iyer, Anil Kumar Gujjari, Sathishbabu Paranthaman, Amr Selim Abu Lila, Khaled Almansour, Farhan Alshammari, El-Sayed Khafagy, Hany H. Arab, Devegowda Vishakante Gowda

**Affiliations:** 1 Department of Prosthodontics, JSS Dental College and Hospital, Mysuru 570015, India; dr.meenakshis@jssuni.edu.in (M.S.I.); dr.anilkumarg@jssuni.edu.in (A.K.G.); 2 Department of Pharmaceutics, JSS College of Pharmacy, Mysuru 570015, India; sathishbabu.p94@gmail.com; 3 Department of Pharmaceutics and Industrial Pharmacy, Faculty of Pharmacy, Zagazig University, Zagazig 44519, Egypt; a.abulila@uoh.edu.sa; 4 Department of Pharmaceutics, College of Pharmacy, University of Hail, Hail 81442, Saudi Arabia; kh.almansour@uoh.edu.sa (K.A.); frh.alshammari@uoh.edu.sa (F.A.); 5 Department of Pharmaceutics, College of Pharmacy, Prince Sattam Bin Abdulaziz University, Al-kharj 11942, Saudi Arabia; e.khafagy@psau.edu.sa; 6 Department of Pharmaceutics and Industrial Pharmacy, Faculty of Pharmacy, Suez Canal University, Ismailia 41552, Egypt; 7 Department of Pharmacology and Toxicology, College of Pharmacy, Taif University, P.O. Box 11099, Taif 21944, Saudi Arabia; h.arab@tu.edu.sa

**Keywords:** *Candida albicans*, clove extracts, cinnamon extracts, denture stomatitis, super critical fluid extraction

## Abstract

Denture stomatitis (DS), usually caused by Candida infection, is one of the common denture-related complications in patients wearing dentures. Clove and cinnamon oils have been acknowledged for their anti-inflammatory, antimicrobial activity, and antifungal effects in the oral cavity. The aim of this study, therefore, was to prepare clove/cinnamon oils-loaded emulgel and to assess its efficacy in treating *Candida albicans*-associated denture stomatitis. Central composite design was adopted to formulate and optimize clove/cinnamon extracts-loaded emulgel. The formulated preparations were assessed for their physical appearance, particle size, viscosity, spreadability, and in-vitro drug release. In addition, in-vivo therapeutic experiments were conducted on 42 patients with denture stomatitis. The prepared emulgel formulations showed good physical characteristics with efficient drug release within 3 h. In addition, in-vivo antifungal studies revealed that the optimized formula significantly (*p* < 0.001) reduced Candida colony counts from the denture surface, compared to commercially available gel (240.38 ± 27.20 vs. 398.19 ± 66.73 CFU/mL, respectively). Furthermore, the optimized formula and succeeded in alleviating denture stomatitis-related inflammation with a better clinical cure rate compared to commercially available gel Collectively, herbal extracts-loaded emulgel might be considered an evolution of polyherbal formulations and might represent a promising alternative to the existing allopathic drugs for the treatment of denture stomatitis, with better taste acceptability and no side effects.

## 1. Introduction

Denture stomatitis is a frequent condition affecting denture wearers that causes inflammation and redness of the oral mucosal tissues covered by the denture [1]. Despite its prevalence, the etiology of denture stomatitis is not completely understood. Nevertheless, denture stomatitis is often associated with Candidal colonization, especially *Candida albicans*, a normal commensal flora of the oral cavity [2]. Along with denture surface colonization, during swallowing and flushing action of saliva, patients can aspire the microorganisms from denture plaque, which could expose patients to unexpected systemic infection especially in immunocompromised patients. Currently, antifungal medications that can combat *Candida albicans* are commonly used to alleviate stomatitis symptoms [2,3]. Nevertheless, despite the availability of an increasing array of over-the-counter anti-fungal drugs, there is an alarming decrease in the success rate of treatment. This might be attributed to the development of anti-fungal resistance, the diverse resistance profile of Candida species, or the lack of patient compliance [4,5]. 

Historically, herbal medicines have a long history and were utilized in ancient Chinese, Egyptian, Greek, and Indian medicine for a variety of therapeutic purposes [6]. According to the World Health Organization, 80 percent of the world’s population still relies mostly on traditional medicines for health treatment. Furthermore, numerous researches have recently demonstrated a paradigm shift from conventional synthetic drugs to natural substances, such as phytochemicals, for the treatment of many diseases, including fungal infections [7,8]. The desirable properties exhibited by phytomedicines are accredited to their phyto-constituents, such as saponins, tannins, alkaloids, flavonoids, terpenoids, and sesquiterpenes, which work in synergy to produce the desired effect. 

For many decades, essential oils and herbal extracts have been employed for various purposes, such as pharmaceuticals, alternative medicine, and natural therapies. Furthermore, polyherbal preparations, containing active substances from one or more herbs, has been repeatedly investigated for their synergistic therapeutic effect [9,10]. When the active phytochemical constituents of individual plants are inadequate to provide the desired therapeutic effects, combining multiple herbs in a certain ratio could significantly enhance the therapeutic effect while minimizing toxicity. Consequently, formulations with the pharmacodynamic synergism of active constituents exhibiting similar therapeutic activity is an added advantage over a single herbal formulation. 

Gels represents a distinct class of semisolid dosage forms that have a wide spectrum of applications for the treatment of various diseases. Gels possess excellent advantages, such as compatibility with various excipients; spreadability; ease of withdrawing; and emollient, non-staining, greaseless, and thixotropic nature [11]. In addition, compared to ointments or creams, gels usually have a higher aqueous component that allows greater dissolution of drugs and permits easy migration of the drug through the vehicle [12]. Nevertheless, despite the many benefits of gels, one significant constraint is the delivery of hydrophobic drugs. To circumvent this limitation, emulsion hydrogels, synonymously called emulgels, were introduced, allowing even hydrophobic drug moieties to benefit from the special features of gels [13,14]. Polymer plays crucial roles in the formulation of emulgels. Because of their gelling capacity, polymers ensure the formulations to be stable by altering the surface and interfacial tension and enhancing the viscosity of the water phase. The gelling agent in the water phase converts typical emulsions into emulgels. Badam gum, a plant exudate obtained from *Terminalia catappa*, is a natural polymer that has been frequently used for the formulation of muco-adhesive drug-delivery systems [15]. Being natural polymer, it exhibits certain advantages over synthetic polymers, namely that it is nontoxic, readily available, economic, and potentially biodegradable.

The aim of this study, therefore, was to formulate clove/cinnamon extracts-loaded emulgels. The prepared emulgel formulations were optimized using central composite design and were analyzed for physical and chemical properties. In addition, the anti-fungal activity of optimized emulgel formulation were assessed clinically against Candida-associated denture stomatitis in geriatric denture wearers.

## 2. Results and Discussion

### 2.1. Percentage Yield of Clove and Cinnamon Extracts

Supercritical fluid extraction (SCFE) technique is the process of extracting one component from a mixture [16]. Several supercritical fluids have been adopted for the extraction of lipid including ethane, ethylene, ethanol, methanol, benzene, and CO_2_ [17]. In this study, CO_2_ was utilized as the supercritical extracting solvent to prepare clove and cinnamon extracts from clove buds and cinnamon barks, respectively. Supercritical carbon dioxide extraction is considered an eco-friendly method where it excludes the use of typical organic solvents [18]. Our results indicated that the percentage yield of clove and cinnamon extracts were 18.9 and 1.9%, respectively, at critical temperature of 37 °C, critical pressure of 300 bar, and an extraction time of 120 min.

### 2.2. Minimum Inhibitory Concentration (MIC) of Clove and Cinnamon Extracts

Many plant products, including plant extract and essential oils, have been acknowledged for their antifungal activities [19,20]. In this study, to assess the antifungal efficacies of clove and cinnamon extracts, the MICs of clove and cinnamon extracts alone and in combination against *C. albicans* isolates were estimated. Both extracts showed antifungal activity against *C. albicans* with MICs of 512 μg/mL for clove extract and 64 μg/mL for cinnamon extract. Similar findings were reported by Stevic et al., who investigated the antifungal potential of 16 selected essential oils against 21 fungi [21]. In addition, the in-vitro interaction of clove and cinnamon extracts against *C. albicans* was examined at concentrations ranged from 32–512 μg/mL for clove extract and 4–64 μg/mL for cinnamon extract (Table S1). The combinations exhibited different interactions against fungal isolates with FICIs ranging from 0.13–2. The combination of clove and cinnamon extracts, at concentrations of 256 and 32 µg/mL, respectively, exhibited additive effect with 99.45% growth inhibition detected at FICI of 1. Most importantly, no regrowth of *C. albicans* was observed upon exposure to clove and cinnamon extracts over 24 h. Of note, combined treatment with clove and cinnamon extracts significantly decreased the MICs values against *C. albicans*, for clove extract from 512 to 256 μg/mL and for cinnamon extract from 64 to 32 μg/mL. Accordingly, combination therapy could be used for expanding the antimicrobial spectrum, reducing toxicity, and decreasing antimicrobial resistance during treatment. 

### 2.3. Solubility and Emulsification Studies

**Figure 1:**
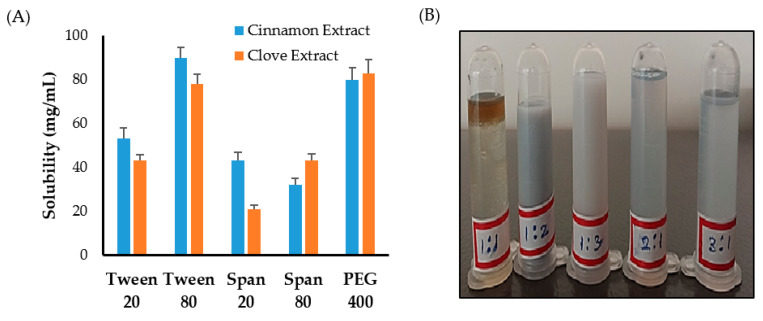
(**A**) Solubility of clove and cinnamon extracts in different surfactants and co-surfactants. (**B**) Emulsion formation from various S_mix_ ratio.

First, solubility studies of clove and cinnamon extracts were conducted in different surfactants and co-surfactants to select the appropriate surfactant/co-surfactant (S_mix_) mixture to be used for emulsification process. As shown in Figure 1A, maximum solubility of clove and cinnamon extracts was observed in Tween 80 and PEG 400 compared to other systems containing Tween 20, Span 20, or Span 80. Accordingly, a surfactant/co-surfactant (S_mix_) mixture composing of Tween 80 and PEG 400 was selected for further emulsification processes.

Next, to study the effect of surfactant mixture (S_mix_) ratio on the emulsification process, different ratios of Tween 80 and PEG 400 were mixed together. It was clear that increasing co-surfactant (PEG 400) concentration from 1:1 to 1:2 or 1:3 resulted in an increase in the turbidity of the system (Figure 1B). On the other hand, increasing surfactant (Tween 80) concentration in surfactant/co-surfactant (S_mix_) mixture from 1:1 to 3:1 while keeping co-surfactant ratio constant resulted in a good and stable emulsions (Figure 1B). Accordingly, the formulation consisting of a minimal S_mix_ ratio (2:1) producing good and stable emulsion was selected for the preparation of emulgel.

### 2.4. Formulation Screening

Quality by design (QbD) is a systematic approach to pharmaceutical development that is commonly used to develop a distinctive product based on pre-determined parameters and studying their effect on certain responses in order to get an optimized formula [22]. In the current study, central composite design (CCD) was adopted as a tool to formulate and to optimize clove/cinnamon extracts-loaded emulgels. A total of 9 runs were prepared, and the effect of two independent variables, namely polymer concentration (% *w*/*w*) and S_mix_ ratio (% *v*/*v*), on the response parameters, namely particle size (nm) and drug content (%), was investigated. In the present design, the optimum formulation was derived by keeping responses of drug content at a maximum level and particle size at a minimum level. The observed responses in CCD of prepared emulgel are summarized in Table 1.

#### 2.4.1. Response Analysis for Optimization of Emulgel

##### Effect of Independent Variables on Globule Size

**Figure 2:**
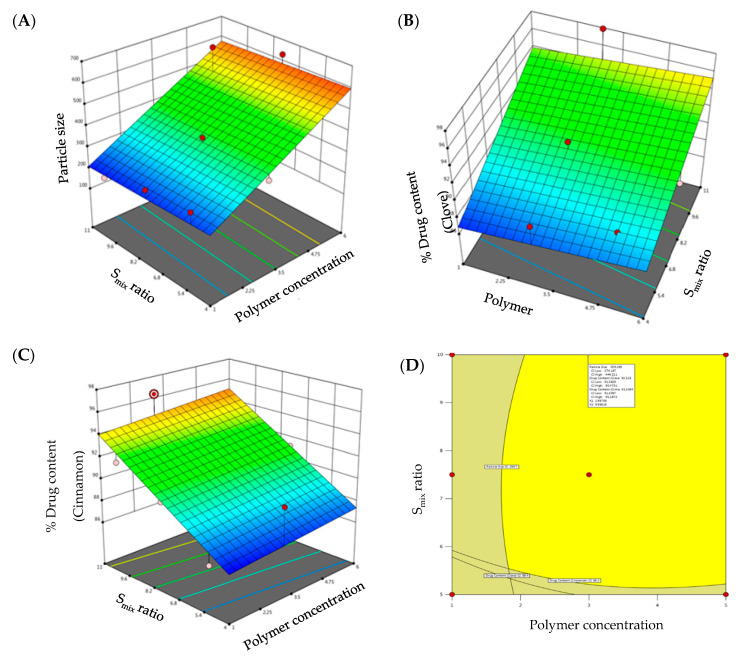
3D-surface plots of (**A**) particle size; (**B**) drug content of clove; (**C**) drug content of cinnamon; and (**D**) overlay plot of optimized emulgel formulation.

Globule size is an important parameter for evaluating the formulated emulgels. The smaller the particle size, the larger the interfacial surface area for drug absorption. Globule size of the developed emulgels was determined, and the results are summarized in Table 1. The globule size of all the formulations was in the range of 198.7 ± 14.7 nm to 631.2 ± 16.4 nm (Table 1). As evident from literature, formulations having particle size <1000 nm are found stable [23]. Hence, the globule size of all the formulations was within the limit. F7 showed the highest particle size, whereas F1 showed the least particle size among the formulations. It was apparent that there is a direct relation between the globule size of the developed emulgels and polymer concentration and S_mix_ ratio. Increment in polymer concentration while keeping S_mix_ ratio constant resulted in a remarkable increase in globule size. On the other hand, increasing S_mix_ ratio while keeping polymer concentration constant resulted in a significant reduction in globule size. Similar results were reported by Chuacharoen et al., who reported that the average particle sizes of curcumin emulsions was decreased by increasing surfactant concentration from 10 to 30% [24]. The impact of polymer concentration and S_mix_ ratio on globule size is depicted by 3D-response surface plot shown in Figure 2A. 

The regression equation verified the effect of formulation variables on globule size. It was obvious that polymer concentration exerted a positive effect on globule size, while S_mix_ ratio exhibited a negative effect on globule size (Equation (1)):

Particle Size (R1) = 165.564 + 74.04 polymer concentration − 2.76 S_mix_ ratio (1)

The obtained F-value of this model was 9.81, and *p* value < 0.0500 implies that the model is significant. 

##### Effect of Independent Variables on Drug Content

Despite the many benefits of gels, one significant constraint is the delivery of hydrophobic drug. To address this constraint, emulgels were created. Drug content of clove and cinnamon in the prepared emulgel formulations was found to range from 86.4 to 97.9% for clove and 86.3 to 96.4% for cinnamon, indicating efficient drug loading within the emulgel. The model F-values of clove drug content (R2) and cinnamon drug content (R3) were 6.28 and 8.28, respectively, implying that the model is significant. The estimated *p*-values were less than 0.05, indicating that the both model terms are significant. Equations (2) and (3), in terms of actual factors, can be used to make predictions about the response for given levels of each factor. As shown in Figure 2B,C, both polymer concentration and S_mix_ ratio displayed a monotonous impact on drug content.

% Drug Content Clove (R2) = 82.51 + 0.269 polymer concentration + 1.02 S_mix_ ratio (2)

% Drug Content Cinnamon (R3) = 81.64 + 0.21 polymer concentration + 1.107 S_mix_ ratio (3)

#### 2.4.2. Optimized Formulation

The CCD design was useful to analyze the obtained polynomial model, which was used to optimize the process of formulating emulgels with fewer formulations. Further, desirability and overlay plots were used to optimize the designed formulations. Optimized formulation was obtained by setting the constraints such that the obtained formulation should have minimal particle size and maximum drug content. Concentrations recommended by DoE to achieve the above-mentioned goals were depicted in the form of an overlay plot (Figure 2D) with desirability near to 1. The optimized formulation, depicted in the overlay plot as a form of a flag, was obtained at a polymer concentration of 2.6% and S_mix_ ratio of 9%. The observed globule size, clove drug content, and cinnamon drug content were 321.2 nm, 96.65%, and 95.94%, respectively, which were close to the predicted values (359.2 nm, 93.52%, and 93.33%) for the optimized formula with a desirability of 1.

### 2.5. Evaluation of Emulgel Formulations 

#### 2.5.1. The Physical Appearance of Emulgel Formulations

Emulgel formulations were white, viscous preparations with homogenous, smooth texture, free from grittiness. The physical appearance, homogeneity, grittiness, and smoothness of all formulations are summarized in Table S2. 

#### 2.5.2. Determination of pH 

The pH of all the formulations was found to be in the range of 5.91 ± 0.09 to 6.72 ± 0.16 (Table 2). There was no substantial change in the pH among all formulations. Accordingly, all the prepared formulations could be considered convenient for oral application.

#### 2.5.3. Measurement of Viscosity

The viscosity values of the prepared gel formulations ranged from 2820.1 ± 0.86 to 7204.9 ± 0.36 (Table 2). Increasing polymer concentration from 1 to 6% significantly increased the viscosity of the formulated emulgel. Similar results are reported by Kotwal et al., who demonstrated that increasing the concentration of poloxamer 407 resulted in a significant increase in the gelling property of the gel [25]. Most importantly, by increasing spindle speed (shear), the viscosity of the gels was decreased. These results stated that all the developed formulations had a non-Newtonian, pseudoplastic, and/or dilatant behavior at a temperature of 25 °C. This type of rheological behavior indicates an appropriate spreadability.

#### 2.5.4. Determination of Spreadability 

The spreading coefficient depends on the polymer concentration in the formulation, and it dictates how easy the emulgel will spread upon applying a small amount of shear. Generally, relatively low value of spreading coefficient enhances emulgel application. All the tested formulae showed good spreadability with a spreadability coefficient ranging from 63.5 ± 0.20 to 90.16 ± 0.09 gm cm s^−1^ (Table 2). 

#### 2.5.5. Extrudability 

Extrudability of the gel from the tube is a very important parameter during application and it affects patient acceptance. Gels with high consistency may not extrude from the tube, whereas low-viscous gels may flow quickly. Consequently, suitable consistency is required to extrude the gel from the tube. The extrudability percentage of different formulations, summarized in Table 3, ranged from 66.43 ± 0.15 to 89.50 ± 0.57, which indicates good to fair extrudability.

#### 2.5.6. In-Vitro Muco-Adhesion 

A significant characteristic of the oral gel is the adhesion to the mucosa. Generally, enhanced gel adhesion results in increased contact time with the mucosa and prolongation of drug contact and clinical efficacy. In-vitro muco-adhesion test revealed that increasing the concentration of polymer-forming gel (badam gum) in F3, F7, and F9 could significantly increase the muco-adhesion of the formulation. Badam gum has a higher molecular weight and could form a large adhesive surface with the mucin and consequently give good muco-adhesiveness [15]. 

#### 2.5.7. FTIR Spectrum

**Figure 3:**
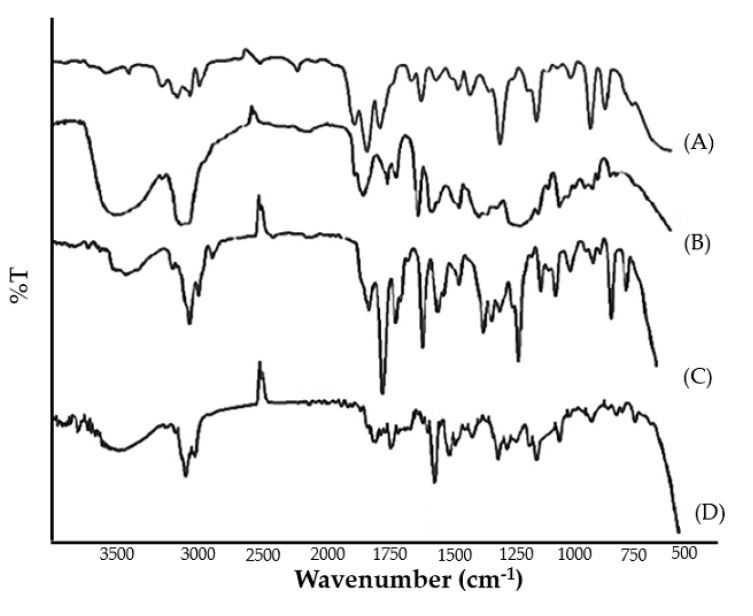
FTIR spectrum of (**A**) pure cinnamon extract, (**B**) pure clove extract, (**C**) physical mixture, and (**D**) clove/cinnamon extracts—loaded emulgel.

FTIR spectra of pure clove and cinnamon extract, physical mixture, and optimized emulgel formulation were recorded. The peaks corresponding to the functional groups of pure clove and cinnamon extract were in correlation with those observed in the FTIR spectra of physical mixture and emulgel formulation (Figure 3). The broad absorption peaks at around 3290 to 3680.30 cm^−1^ were assigned to the phenolic OH stretching vibration. The absorption peaks positioned at 1612 cm^−1^, 1516 cm^−1^, 1429 cm^−1^, and 1240 cm^−1^ are assigned to the C-C, C=O, C=C (aromatic), and C-O (aryl ether), respectively. From the results, it was evident that no chemical interactions were observed between pure clove and cinnamon extract and its physical mixture and emulgel formulation. Therefore, it can be inferred that the selected pure clove and cinnamon extract was compatible with the selected S_mix_ and polymer.

### 2.6. In-Vitro Drug Release Studies 

**Figure 4:**
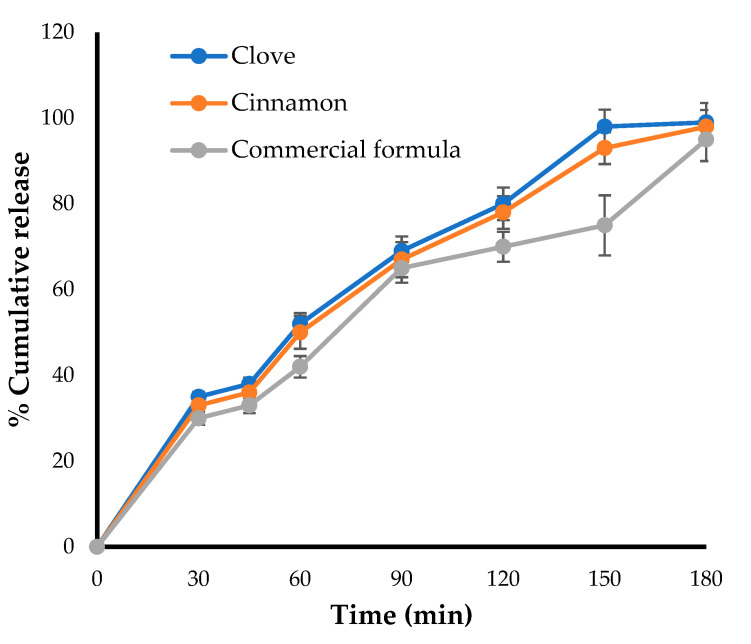
Drug release profile for optimized emulgel formula and marketed (Candid^®^) gel.

The in-vitro release pattern was carried out for the optimized formulation and was compared with that of a marketed preparation using Franz diffusion cell. As shown in Figure 4, the amount of drugs (clove and cinnamon) released from the optimized formula was comparable to the amount of clotrimazole released from the marketed gel formulation, with about 50% of the loaded drug released within 50 min. In addition, nearly 100% of loaded drugs were released from both formulae after 180 min, suggesting the efficient release of clove and cinnamon extracts from the optimized formula and nullifying the possible detrimental effect of gel-forming polymer on drug release from emulgel formulation.

### 2.7. Stability Studies

Stability testing was conducted for the optimized emulgel formulation at different storage conditions, and the results are summarized in Table S3. It was obvious that non-significant changes were detected in the physical appearance, pH, and drug content of the stored emulgel formulation over one and three months compared to fresh preparation (*p* > 0.05). The results confirmed the stability of the formulated clove/cinnamon extracts-loaded emulgel.

### 2.8. Randomized Clinical Trial

#### 2.8.1. Study Design

A randomized double-blind controlled study was adopted to assess and compare the antifungal potential of clove/cinnamon extracts-loaded emulgel with a commercially available antifungal gel (Candid^®^) for the treatment of denture stomatitis. A total of 42 volunteers were randomly divided into two groups: one receiving the optimized emulgel formulation and the second receiving Candid^®^ gel. The mean ages of test and control groups were 61.57 ± 7.8 and 62.86 ± 6.5, respectively. The CONSORT Flowchart is depicted in Figure S1. Both groups presented male predominance, and the chi-square test revealed no significant association between age or gender among both the groups (Table 3). In addition, although denture age was not related to the degree of inflammation in both the test (*p* = 0.334) and control (*p* = 0.156) groups, it was observed that 66.7% of patients in the test group and 53.3% patients in the control group had Newton’s Type II inflammation. Complete dentures with full palatal coverage could encourage Candidal colonization due to an increase in an acrylic volume covering the palatal surface, which could provide a large area for adhesion of microorganisms and decreased cleansing action by saliva and tongue. Accordingly, there was an association between the duration of denture wear and the occurrence and/or progression of denture stomatitis inflammation stage. Furthermore, 53.8% of patients in the test group and 42.9% patients in the control group who practiced nocturnal denture wear showed Newton’s Type II denture stomatitis.

#### 2.8.2. Antifungal Efficacy of Clove/Cinnamon Extracts-Loaded Emulgel 

**Figure 5:**
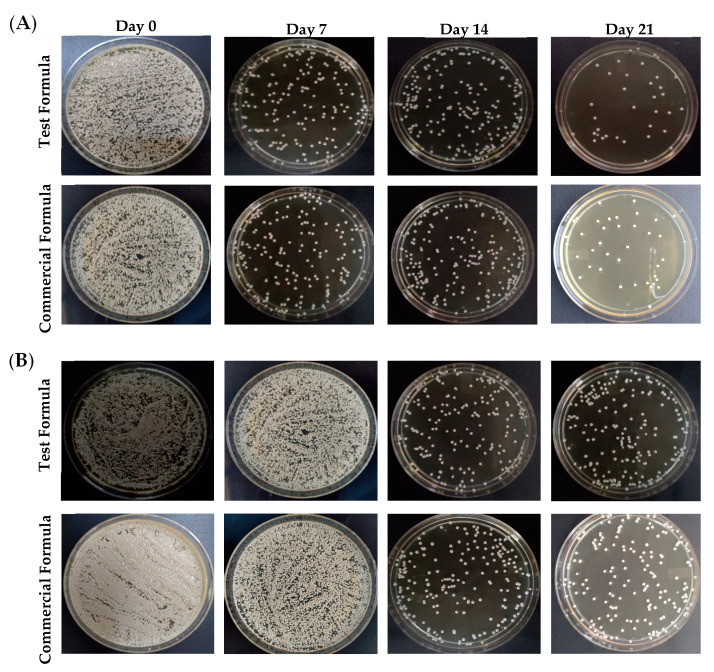
Mean fungal growth reduction (CFU/ mL) from the (**A**) palatal surface and (**B**) denture surface upon treatment with either test formula or a commercially available gel on days 0, 7, 14, and 21 post treatment.

To assess the antifungal efficacy of optimized formula of clove/cinnamon extracts-loaded emulgel, patients were treated with either the optimized formula (Group A) or a commercially available marketed gel (Candid^®^; Group B) on days 7, 14, and 21 after the first visit. Swab samples were collected from palatal and denture surface at baseline and after the intervention at 7, 14, and 21 days. Comparisons of the microbial growth, pre-, and post-intervention in terms of colony-forming units (CFU per mg of plaque) were assessed for each group. As depicted in Figure 5, treatment with either the test formula or the commercially available gel (Candid^®^) significantly reduced microbial growth on both palatal and denture surfaces compared to control (untreated) group. In addition, such reduction of microbial growth in both groups was significantly correlated with the frequency of dosing; microbial growth inhibition was much more observed in both treated groups on day 21 post treatment following three treatments as compared to microbial growth on days 7 or 14 post intervention (Table 4). The microbial growth on palatal and denture surface post-test formula application were 91.76 ± 61 CFU/mL and 240.38 ± 27 CFU/mL, respectively, on day 21 post intervention as compared to 432.8 ± 236 CFU/mL and 815.8 ± 145 CFU/mL on day 7 post intervention, respectively. 

Next, intergroup comparison were conducted to compare the antifungal efficiency of test formula (Group A) with that of a commercially available gel (Candid^®^; Group B). The percentage reduction of colony-forming units (CFU) of test formulation and commercially available marketed gel groups was calculated and was used as an assessment parameter for comparison. As summarized in Table 5, both test and commercial formulations efficiently suppressed microbial growth from palatal and denture surfaces in a dose frequency-dependent manner. The percentage reduction of microbial growth on both palatal and denture surfaces for test formula was much higher at day 21 post treatment (three sequential treatments) compared to those observed on either day 7 (one treatment) or day 14 (two sequential treatments). Most importantly, the percentage reduction of CFU, on day 21 post treatment, on denture surface of test formulation was superior to that of the commercially available marketed gel. Collectively, these results emphasized the efficient anti-fungal potential of test formula against Candida species.

#### 2.8.3. Clinical Response to Treatment with Clove/Cinnamon Extracts-Loaded Emulgel

**Figure 6:**
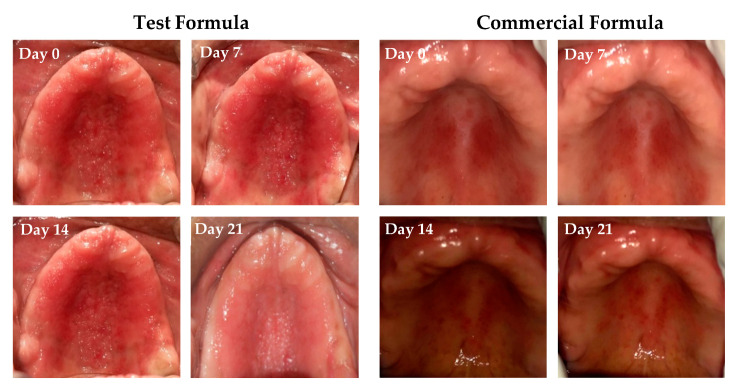
Pictorial representation of clinical response to treatment of Group A and B on Stage III and Stage II conditions on days 0, 7, 14, and 21 post treatment.

The response to treatment with either clove/cinnamon extracts-loaded emulgel or the commercially available gel (Candid^®^) in patients suffering from denture stomatitis was evaluated clinically. As depicted in Figure 6, treatment with optimized emulgel formula significantly enhanced the clinical outcomes in patients suffering from denture stomatitis as manifested by a remarkable improvement in erythema of palatal mucosa. In addition, as summarized in Table 6, treatment with optimized test formula significantly alleviated denture stomatitis-related inflammation, compared to Candid^®^-treated group, in a treatment duration-dependent manner. The complete cure rate percentage in patients treated with the optimized formula increased from 19% on day 7 post treatment to 47.6% on day 21 post treatment. Most importantly, there was a remarkable shift from Newton’s stages II/III denture stomatitis to Newton’s stage I denture stomatitis, and no patients suffering from either Newton’s stage II/III denture stomatitis were observed on day 21 post treatment. On the other hand, more than 20% of Candid^®^-treated group still suffered from Newton’s stage II denture stomatitis even following three weeks’ treatment. These results emphasized that the optimized emulgel formula could provide superior clinical remission of Newton’s classification and reduction of fungal burden compared to the commercially tested gel (Candid^®^). Chi-square test was conducted for test and control groups to evaluate the influence of applied treatment on clinical improvement and found statistically significant improvement in test group when compared to the control group (Table S4).

#### 2.8.4. Trial Adherence

To address treatment adherence, dosing compliance was calculated by weighing the tubes at baseline and one week, two weeks, and three weeks of the usage of gels. Patients who consumed more than 5 g of gel during treatment were considered adherents [26]. Greater adherence was shown for the test group, where the mean usage of gel was 7.83 ± 0.85 g at 7 days post usage, 7.63 ± 0.54 g at 14 days post usage, and 5.73 ± 0.738 g at 21 days post usage. On the other hand, commercial gel usage was 5.78 ± 0.79 g, 5.82 ± 0.93 g, and 5.04 ± 0.64 g at days 7, 14, and 21 post usage, respectively. Based on these results, we expect a high degree of compliance to the optimized test formula.

#### 2.8.5. Taste Acceptability

Taste acceptability represents one of the crucial parameters that might affect patience compliance for the treatment. In the study, based on the Hedonic scale [27], 81% of investigated patients in the test group showed greater likeliness for the formulated gel. By contrary, 76.2% of patients treated with marketed gel disliked the gel. This result indicates the greater taste acceptability of optimized emulgel formula compared to marketed gel. This might explain with the improved treatment adherence in patients treated with test formula compared to marketed product.

Although the key factor in the treatment of chronic infection such as denture stomatitis is efficient denture cleaning, the denture cleansing action and mechanism of biofilm regrowth seems unclear [28,29]. In this study, most of the patients used to clean their dentures with soap and brush, but denture stomatitis was still detected, which concluded that only mechanical brushing does not cure denture stomatitis [30]. Many commercially available cleansers are ineffective at inhibiting Candida biofilms [29]. With the global threat of antimicrobial resistance, a paradigm shift to research into plant extracts and/or plants essential oils has been revisited. The essential oil targets multiple microbes, thus exhibiting broad-spectrum antimicrobial activity with little or no occurrence of antimicrobial resistance [31,32]. In this study, we revealed for the first time the efficacy of clove oil and cinnamon bark oil incorporated polyherbal emulgel treatment for *Candida albicans*-associated denture stomatitis. 

## 3. Conclusions

In this study, we succeeded to develop and optimize clove and cinnamon supercritical fluid extracts-loaded emulgel and compared its antifungal potential in denture stomatitis with a commercially available marketed product. The inter-group comparison between the two formulations inferred a comparable reduction in CFU percentage from the palatal surface between both groups. In addition, both formulations efficiently alleviated denture stomatitis-related inflammation in a treatment duration-dependent manner with comparable clinical cure rates. Accordingly, herbal extracts-loaded emulgel might be considered an evolution of indigenous polyherbal formulations and might represent a promising alternative to the existing allopathic drugs for the treatment of denture stomatitis, with better taste acceptability and no side effects.

## 4. Materials and Methods

### 4.1. Materials 

Clove bud, cinnamon bark, and almond gum were purchased from the Govindaraja Shetty and Sons (Mysuru, India). Clotrimazole was procured from Sigma Aldrich (St. Louis, MO, USA). The freeze-dried form of the microorganism *Candida Albicans* (ATCC 10231) and human gingival fibroblast (HGF) (ATCC^®^PCS-201-018TM) were obtained from American Type Culture Collection. Fetal Bovine Serum and Dulbecco’s modified eagle medium (DMEM) were purchased from Gibco (Fort Worth, TX, USA). All other solvents and reagents were of analytical grade. 

### 4.2. Preparation of Supercritical Fluid Plant Extract 

All the procured and authenticated clove buds and cinnamon barks were dried in shade, cleaned by hand, sorted, and powdered. A weight of 10 kg of powered individual herbs were then loaded in the supercritical fluid (SCF) extractor unit. SCF extraction was done using CO_2_ gas without any co-solvents. Supercritical CO_2_ was fed to the extractor through a high-pressure pump (300 bar) at 37 ± 0.5 °C, which was above its critical temperature and pressure. The extract-laden CO_2_ was then sent to a separator (60–120 bar) via a pressure-reduction valve. The temperature and pressure were reduced so that the extract precipitated into the separator, and gaseous CO_2_ was released into the atmosphere [33]. 

### 4.3. Quantification of Herbal Extracts Constituents by Gas Chromatography 

Gas chromatography (GC; Shimadzu GC- 2014, Tokyo, Japan) with a flame ionization detector was used for the detection of standard active compounds (Eugenol, Cinnamaldehyde, and benzaldehyde) in the herbal extracts. Gas chromatograph was equipped with an Rtx^®^-5 fused-silica column (30-m 0.25-mm id, film thickness 0.25 µm). The oven temperature of GC was programmed to an increasing temperature from 80 to 230 °C at a time rate of 6 °C/min and at 230 °C for 2 min. Detector and injector temperatures were set at 230 °C. The helium and nitrogen were adjusted at a linear velocity of 24 mL/min. A total of 1 µL of the samples (clove and cinnamon extracts dissolved in HPLC-grade chloroform) were injected into the GC using split mode with a split ratio of 10 to 1. Stock solutions were prepared by precisely weighing 10 mg of eugenol, cinnamaldehyde, or benzaldehyde and dissolving them in 10 mL of HPLC grade chloroform. A series of aliquots (2 to 10 µg/mL of eugenol, cinnamaldehyde, or benzaldehyde) were prepared by serially diluting stock solution and was quantified with GC to construct standard calibration curves [34,35].

### 4.4. Antifungal Activity of the Herbal Extracts 

The minimum inhibitory concentrations (MICs) of either clove or cinnamon extracts alone or in combination against *C. albicans* isolates were determined with a broth microdilution method as described by the CLSI guidelines [36]. Briefly, in a 96-well plates, 100 μL of serial dilutions of clove extract, cinnamon extract alone or clove/cinnamon extracts, having the concentrations of 512, 256, 128, 64, 32, 16, 8, 4, 2, and 1 µg/mL, were added to each well. A drug-free well served as a negative control. Then, 100 μL aliquots of inoculum of the test strain, *C. Albicans*, adjusted to 1.5 × 10^6^ CFU/mL equal to 0.5 McFarland, were taken aseptically and then added to each well and kept for incubation at 35 ± 2 °C under anaerobic conditions for 24 h. After the specified incubation time, the growth inhibition was determined both by visual inspection.

The in-vitro interaction of clove and cinnamon extracts against *C. Albicans* was interpreted in terms of the fractional inhibitory concentration index (FICI) as follows [37]: 

FICI = FIC_clove_ + FIC_cinnamon_ = (MIC of clove in combination/MIC of clove alone) + (MIC of cinnamon in combination/MIC of cinnamon alone).

The interpretation of the FICI was defined as FICI of ≤ 0.5 for synergy, 0.5 < FICI ≤ 1.0 for additive effect, and FICI > 1.0 for antagonism. 

### 4.5. Solubility and Emulsification Studies

The solubility of clove and cinnamon extracts was determined in various surfactants (Tween 20, Tween 80, Span 20, and Span 80) and co-surfactant (PEG 400) by adding an excess amount of extract in 1 mL of the selected vehicle. In a typical procedure, an excess of the drug (clove and cinnamon extracts) was mixed in the respective systems using a vortex mixer. The mixtures obtained were set aside for 72 h and centrifuged for 10 min at 5000 rpm. Then, 0.5 mL of supernatant was drawn out, diluted, and analyzed for both clove and cinnamon solubility using GC as aforementioned. 

To select the best suitable ratio of surfactant and co-surfactant (S_mix_ ratio) to obtain the desired emulgel, emulsification studies were performed. Tween 80 and PEG 400 were selected as surfactant and co-surfactant, respectively. Five different S_mix_ ratios were evaluated (1:1, 1:2, 1:3, 2:1, and 3:1) for the emulsifying ability. The obtained solutions were mixed using a vortex mixer to form homogenous blends.

### 4.6. Preparation of Emulgel

#### 4.6.1. Experimental Design

To demonstrate the response surface model by attaining different combination of values, design expert software (Version 12, Stat-Ease Inc. and Minneapolis, MN, USA) was employed. A 2-factor, 2-level central composite design was used to design the optimized procedure to formulate various emulgel formulations and to investigate the impact of two formulation variables, namely polymer concentration and surfactant/co-surfactant (S_mix_) ratio on formulations parameters, namely particle size AN drug content. The experimental design matrix of the central composite design is summarized in Table 7. A total 9 runs were performed to obtain the optimized formula and to achieve the desired responses.

#### 4.6.2. Formulation of Clove/Cinnamon Extracts-Loaded Emulgel

Different formulations were prepared using varying amounts of gel-forming polymer (badam gum; 1–6% *w*/*w*) and different ratios of surfactant/co-surfactant mixture (S_mix_; 5–10% *v*/*v*). Briefly, specified weighs of clove and cinnamon extracts at a ratio of 1:8, corresponding to their MIC values, were mixed with different ratios of Tween 80 and PEG 400 (S_mix_) mixture, forming an oily phase. Varying amounts of gel-forming polymer (badam gum) were soaked in water and served as the aqueous phase. The oily phase was added to the aqueous phase portion wise and was homogenized at 15,000 rpm for 15 min to form different emulgel formulations [11].

### 4.7. Evaluation of Clove/Cinnamon Extracts-Loaded Emulgel

#### 4.7.1. Physical Examination

Appearance, color, homogeneity, grittiness, and smoothness of the prepared emulgel formulations were tested both visually and by touch.

#### 4.7.2. Determination of pH 

The pH of different emulgel formulation was measured by standardized pH meter (MW802, Milwaukee Instruments, Szeged, Hungary). The pH measurements were performed in triplicates, and the mean value was calculated.

#### 4.7.3. Viscosity Measurement

The viscosity (in cPs) of the formulated emulgels was determined by Brookfield viscometer using spindle number T-F at 25 °C. The viscosity measurements were performed in triplicates, and the mean value was tabulated. 

#### 4.7.4. Determination of Spreadability

As per the International standards for Harmonization (ICH) guidelines, 1 g of emulgel sample was sandwiched in between two glass plates (20 cm × 20 cm). A definite load (500 g) was applied on the upper glass plate for 1 min. Later, the radius of the spread gel was measured. The spreadability (g cm/s) was measured by using the following equation:

S = (m × l)/t

where S is the spreadability (g cm/s), m is the mass of the weight applied (gm), l is the radius of the spread gel (cm), and t is the time taken (s).

#### 4.7.5. Extrudability

The emulgel formulations were placed in standard capped collapsible aluminum tubes crimped at the end, and the weights of each tube was recorded. The tubes were placed between two glass slides and were clamped. A definite weight of 500 g was placed over the slides, and the amount of the emulgel extruded from tubes was weighed [38]. The percent of the extruded gel was calculated, and the extrudability is recognized as excellent if extrudability > 90%, good if extrudability > 80%, and fair if extrudability > 70%.

#### 4.7.6. Determination of Globule Size of Emulgel Formulations 

The globule size of all emulgel formulations was measured by the internal light scattering technique using a Malvern instrument (Malvern Nano ZS90, Malvern Instrument Ltd., Worcestershire, UK). To avoid multiple scattering, concentrated emulsions were diluted in (1:1000) with deionized water before analysis. The droplet size distribution of each gel was measured trice, and the mean droplet diameter was reported as the average.

#### 4.7.7. Drug Content Study 

The drug content of all the formulations was measured by dissolving an accurately weighing 100 mg of gel formulations in 10 mL of HPLC-grade chloroform. The solution was filtered through 0.45-mm membrane filter, and further serial dilutions were made and subjected for drug content uniformity by using gas chromatography. 

#### 4.7.8. In-Vitro Muco-adhesion Measurement 

In-vitro muco-adhesion for emulgel formulations was evaluated by a modified tensiometer technique adopted from Fisher’s tensiometer [39]. Briefly, 200 mg of emulgel were taken on a mica disk and were placed on the tensiometer, allowing the gel to come in contact with 1% (*w*/*v*) sodium alginate solution for 5 min. Later, the gel was removed from the solution of sodium alginate at 0.2 inches/min rate. The adhesion force between the mica disk and the sodium alginate solution was employed as the blank. The detachment force was calculated in terms of dyne/cm^2^, and the study was performed in triplicates.

#### 4.7.9. Fourier-Transform Infrared (FTIR) Analysis

Fourier-transform infrared (FTIR) spectra of both pure extract, physical mixtures of clove and cinnamon, and emulgel were recorded using Shimadzu 8400S spectrometer (Tokyo, Japan) to evaluate the compatibility between the selected extracts and S_mix_. A physical mixture was prepared by employing the sodium chloride (NaCl) plate method, where (NaCl) was used to hold samples and scanned at a resolution of 4 cm^−1^ in the range of 4000–400 cm^−1^. 

### 4.8. In-Vitro Drug Release Studies 

The in-vitro drug release studies were conducted using a Franz diffusion cell. Briefly, cellophane membrane (12,000–14,000 MW) previously soaked in a phosphate buffer of pH 7.4, was fixed in between donor and receiver cells. Then, 100 mg of the optimized emulgel formulation or a commercially available gel (Candid^®^) were placed into donor compartment. Phosphate buffer pH 7.4 was used as a dissolution media. The temperature of the cell was kept at 37 ± 0.5 °C by circulating water jacket, and the buffer was stirred continuously at 100 rpm. At definite time points (0, 15, 30, 45, 60, 90, 120, 150, and 180 min), aliquot samples were collected and were replaced with equal volumes of fresh medium to maintain the sink condition. The collected samples containing clove and cinnamon were extracted by chloroform, filtered through a 0.45-mm membrane filter, further diluted, and finally analyzed by gas chromatography as aforementioned. The amount of clotrimazole released from Candid^®^ gel was determined by HPLC as previously described [40]. Briefly, HPLC (Shimadzu, Kyoto, Japan) equipped with C18 (150 mm × 4.6 mm, 5 μm) column was used. The mobile phase consists of acetonitrile and water (70:30% *v*/*v*). The flow rate was 1.0 mL/min, and the UV detection was set at 210 nm. Clotrimazole was eluted at 5.6 min. Clotrimazole concentration was estimated from a pre-constructed calibration curve of clotrimazole at various concentrations. 

### 4.9. Stability Studies

The stability study was carried out in accordance with ICH guidelines. Briefly, the optimized emulgel formulation was packed in collapsible tubes and kept for three months at varied temperature and humidity settings, namely 25 ± 2 °C and 40 ± 2°C and 65% RH, for 3 months and was then tested for various parameters, such as appearance, pH, and drug content.

### 4.10. Randomized Clinical Trial 

#### 4.10.1. Ethical clearance

The study proposal was submitted for approval and clearance was obtained from the ethical committee (No. ECR/1170/inst/KA/2019), JSS Dental College and Hospital under JSS AHER, Mysuru, India.

#### 4.10.2. Study Design and Study Setting 

A randomized, double-blind, controlled clinical trial, conducted at department of Prosthodontics, JSS Dental College and Hospital, was adopted to evaluate the antifungal potential of optimized clove/cinnamon extracts-loaded emulgel against Candida species and to compare it with a commercially available gel in patients with denture stomatitis. Microbial swabs from selected patients with denture stomatitis were collected in the department of Prosthodontics, JSS Dental College and Hospital, and the microbial testing was carried out at department of Microbiology, JSS College of Life Sciences, Mysuru, India. All data regarding estimation of sample size, selection criteria, and randomization are provided as supplementary materials.

#### 4.10.3. Biological Sample Collection and Analysis

Participants with more than 50 colonies were considered positive for fungal infection and were subjected to biological sample collection and analysis. For biological sample collection, a swab stick (ETO, sterile) was used to collect biofilms from the oral palatal and denture surfaces. Swabs were mixed separately with saline diluted according to the defined protocol. The swabs were immediately transferred to the Microbiology Department, College of Life Sciences, for plating and culture analysis. Briefly, 50 µL of the inoculum (1/100 dilution sample) were pipetted out and were plated onto Sabouraud dextrose agar (SDA) plates supplemented with chloramphenicol (0.1 g/mL medium) and incubated at 37 °C for 48 h. The procedure was done inside a laminar airflow chamber to ensure an even distribution of the sample. 

#### 4.10.4. Interventions and Blinding

A total of 42 patients, divided into two groups, were subjected to this study. The tubes with the medicaments were placed in a container and randomly divided and numbered into two groups: Group A (Optimized emulgel) and Group B (Commercially available gel (Candid^®^ gel)). The tubes were labeled using a PVC stick-on label with coding and were segregated according to grouping. The tubes were then distributed as per participant’s number and group. Verbal instructions and a written protocol were given to the volunteers in each study group for ensuring the appropriate application of gels under test. 

To evaluate the fungal burden, on days 7, 14, and 21 after the first visit, oral palatal and denture swab samples were obtained and were assessed for the number of colony-forming units per milliliter (CFU/mL) using colony count software (Image J) (Figure S2). Briefly, digital photographs of the cultures were captured and analyzed for fungal colonies per swab (CFU/swab) using Open CFU software. The number of fungal CFU/mL was calculated by multiplying the number of colonies in each sample by the dilution factor [41]. The clinical and mycological examination were conducted before and after treatment, and the clinical effectiveness at each stage of treatment was correlated to clinical alterations in the severity and reduction in the colony counts. 

#### 4.10.5. Treatment Adherence

The sequentially numbered tubes with the medicaments were weighed before the study and on the 7th, 14th, and 21st days to estimate the patient adherence to treatment. Participants using more than 5 g gel throughout the study period were recognized as adherents. The appointments of the participants were arranged to avoid communication between participants to minimize research outcome bias. 

#### 4.10.6. Taste Acceptability

Subjective evaluation of the participants was conducted to analyze taste acceptability of products under investigation, using a Hedonic scale adapted by Gacula et al. [27], which ranged from liked extremely to dislike very much.

### 4.11. Statistical Analysis

The paired *t*-test was used to evaluate the reduction of fungal burden in both Group A and Group B. The CFU/mL reduction was analyzed by repeated measure ANOVA. Differences between treatment groups in clinical cure and improvement rates were tested for significance by the chi-square test. The acceptability of the products was compared using the Mann–Whitney test and Crammers for the association. All the analysis was performed using SPSS version 18.0 software. A value of *p* < 0.05 was considered statistically significant.

## Figures and Tables

**Table 1:** Observed responses in central composite design of Emulgel.

Formulation Code	X1 Polymer Conc. (% *w*/*w*)	X2 S_mix_ Ratio (% *v*/*v*)	R1 Globule Size (nm)	R2 Drug Content Clove (%)	R3 Drug Content Cinnamon (%)
F1	1	10	198.0 ± 14.7	91.6 ± 0.23	92.1 ± 0.12
F2	3	11	224.2 ± 12.3	97.9 ± 0.09	96.4 ± 0.24
F3	5	10	624.1 ± 12.5	92.5 ± 0.17	93.7 ± 0.12
F4	3	4	347.7 ± 14.1	89.6 ± 0.22	90.1 ± 0.14
F5	1	5	280.4 ± 14.5	86.4 ± 0.15	86.3 ± 0.28
F6	3	7.5	387.9 ± 12.7	91.8 ± 0.31	89.2 ± 0.23
F7	6	7.5	631.2 ± 16.4	90.4 ± 0.32	91.2 ± 0.16
F8	1	7.5	258.8 ± 8.4	89.8 ± 0.12	90.1 ± 0.15
F9	5	5	424.1 ± 10.3	89.1 ± 0.20	86.4 ± 0.34

All data represent the mean ± SD of three independent experiments.

**Table 2:** Physicochemical parameters of clove/cinnamon extracts-loaded emulgel formulations.

Formulation Code	pH	Viscosity (cPs)	Spreadability (g cm/s)	Extrudability (%)	Muco-adhesion (dyne/cm^2^)
F1	6.23 ± 0.12	2820.1 ± 0.86	90.16 ± 0.09	84.26 ± 0.07	5.8 ± 0.14
F2	6.26 ± 0.06	4090.2 ± 0.21	89.03 ± 0.19	79.50 ± 0.15	8.5 ± 0.04
F3	6.72 ± 0.16	6224.8 ± 0.13	81.16 ± 0.09	89.50 ± 0.57	13.9 ± 0.07
F4	6.23 ± 0.06	4012.3 ± 0.62	87.67 ± 0.08	78.53 ± 0.15	8.0 ± 0.11
F5	6.50 ± 0.10	4040.0 ± 0.53	86.83 ± 0.37	77.00 ± 0.06	10.2 ± 0.15
F6	6.43 ± 0.15	4204.9 ± 0.36	63.5 ± 0.20	82.33 ± 0.57	10.4 ± 0.04
F7	5.91 ± 0.09	7204.9 ± 0.36	76.33 ± 0.07	66.43 ± 0.15	15.4 ± 0.74
F8	6.33 ± 0.05	4010.0 ± 0.33	84.33 ± 0.17	74.50 ± 0.57	9.7 ± 0.42
F9	5.91 ± 0.09	6426.9 ± 0.39	76.33 ± 0.07	86.50 ± 0.57	13.9 ± 0.07

Data represent mean ± SD of three independent experiments.

**Table 3:** Demographic details of study participants.

Variable				Group			Total	Test Statistics		*p*-Value	
				Test		Control					
Mean age				61.57 ± 7.8		62.86 ± 6.5	-	t = 0.58		0.564	
Age groups (years)	<50		Frequency	3		1	4	X^2^ = 1.333		0.721	
			Percent	14.3		4.8	9.5				
	51–60		Frequency	5		7	12				
			Percent	23.8		33.3	28.6				
	61–70		Frequency	10		10	20				
			Percent	47.6		47.6	47.6				
	70+		Frequency	3		3	6				
			Percent	14.3		14.3	14.3				
Gender	Male		Frequency	17		18	35	X^2^ = 0.155		0.694	
			Percent	81.0		85.7	83.3				
	Female		Frequency	4		3	7				
			Percent	19		14.3	16.7				
Use of denture (years)	<5		Frequency	5		4	9	X^2^ = 10.48		0.005	
			Percent	23.8		19.0	21.4				
	>5		Frequency	16		17	33				
			Percent	76.2		21	78.6				
Inflammation stages	**Test Group (A)**					**Control Group (B)**				***p*-Value**	
	**Denture Age**	**I**	**II**	**III**	**Total**	**I**	**II**	**III**	**Total**	**A**	**B**
	<5	50.0%	50.0%	0.0%	100.0%	33.3%	50.0%	16.7%	100.0%	0.334	0.156
	>5	20.0%	66.7%	13.3%	100.0%	40.0%	53.3%	6.7%	100.0%		
**Variable**				**Test Group (A)**			**Control Group (B)**			***p* value**	
				**YES**	**NO**	**Total**	**YES**	**NO**	**Total**	**A**	**B**
Nocturnal denture wear	Type of DS	I	Count	6	0	6	8	0	8	0.590	0.645
				(46.2%)	(0.0%)	(28.6%)	(57.1%)	(0.0%)	(38.1%)		
		II	Count	7	6	13	6	5	11		
				(53.8%)	(75.0%)	(61.9%)	(42.9%)	(71.4%)	(52.4%)		
		III	Count	0	2	2	0	2	2		
				(0.0%)	(25.0%)	(9.5%)	(0.0%)	(28.6%)	(9.5%)		

**Table 4:** Pre-intervention and post-intervention microbial growth with Group A and Group B from palatal and denture surface ranges.

Test Group	Group A		Group B	
	Palatal Surface	Denture Surface	Palatal Surface	Denture Surface
**Baseline ** **(Day 0)**	649.71 ± 282.82	1092.04 ± 150.12	677.38 ± 303.21	1160.76 ± 194.72
**Post Treatment**				
**Day 7**	432.80 ± 236.42	815.80 ± 145.04	449.80 ± 211.81	815.23 ± 95.4328
**Day 14**	228.85 ± 136.85	572.42 ± 151.42	231.61 ± 115.77	568.66 ± 136.85
**Day 21**	91.76 ± 61.07	240.38 ± 27.20	66.38 ± 39.11	398.19 ± 66.73

**Table 5:** Percentage reduction of CFU in Group A and B.

Day	Reduction of CFU in Percentage			
	Palatal Surface		Denture Surface	
	Group A	Group B	Group A	Group B
Baseline vs. 7 days	33.38%	33.59%	25.2%	27.06%
Baseline vs. 14 days	64.78%	64.80%	47.58%	49.36%
Baseline vs. 21 days	85.9%	90.20%	77.98%	71.68%
7 days vs. 14 days	47.2%	48.51%	29.80%	30.03%
14 days vs. 21 days	59.9%	71.34%	70.05%	44.03%
7 days vs. 21 days	78.7%	85.2%	58.07%	30.80%

**Table 6:** Clinical response to treatment between test and control groups.

Group	Duration	Newton’s Classification			
		I	II	III	Cure
Group A (Test formula)	0 (baseline)	6	13	2	0
		(18.8%)	(44.8%)	(100.0%)	(0.0%)
	7 days	6	11	0	4
		(18.8%)	(37.9%)	(0.0%)	(19.0%)
	14 days	9	5	0	7
		(28.1%)	(17.2%)	(0.0%)	(33.3%)
	21 days	11	0	0	10
		(34.4%)	(0.0%)	(0.0%)	(47.6%)
Group B (Candid^®^)	0 (baseline)	9	10	2	0
		(22.5%)	(47.6%)	(100.0%)	(0.0%)
	7 days	13	5	0	3
		(32.5%)	(23.8%)	(0.0%)	(14.3%)
	14 days	11	1	0	9
		(27.5%)	(4.8%)	(0.0%)	(42.9%)
	21 days	7	5	0	9
		(17.5%)	(23.8%)	(0.0%)	(42.9%)

**Table 7:** Variables in center-composite design for preparation and optimization of emulgel.

Factors	Levels	
Independent Variable	Low	High
X_1_ = Polymer (% *w*/*w*)	1	6
X_2_ = S_mix_ (% *v*/*v*)	4	11
**Dependent variable**	**Goals**	
R_1_ = Globule size (nm)	Decrease	
R_2_ = Drug Content clove (%)	Increase	
R_2_ = Drug Content cinnamon (%)	Increase	

## Data Availability

The data presented in this study are available on request from the Corresponding author.

## Supplementary Materials

The following supporting information can be downloaded at: https://www.mdpi.com/article/10.3390/gels8010033/s1, Data S1: Estimation of sample size, selectin criteria and randomization; Figure S1: CONSORT Chart, Figure S2: Colony counting on SDA by using Colony Count Software, Table S1: Drug interactions of clove and cinnamon extracts against *C. albicans* in vitro, Table S2: Physical appearance of Emulgel; Table S3: Stability data of clove/cinnamon extracts-loaded emulgel at 25 ± 2 °C and 40 ± 2 °C, Table S3: Chi-Square tests of Clinical response to treatment between both the groups.
