# Supplementary material for: Development and Evaluation of Clove and Cinnamon Supercritical Fluid Extracts-Loaded Emulgel for Antifungal Activity in Denture Stomatitis
Source: Gels. 2022 Jan 4;8(1):33. doi: 10.3390/gels8010033 (PMC8774589; doi:10.3390/gels8010033)
Supplement: Supplementary file 1 [file gels-08-00033-s001.zip › gels-1533478-supplementary.pdf]

Supplementary Materials

Sample Size

Sample size calculations allow researchers to make strong, reliable conclusions from a relatively limited amount of information while also allowing results to be generalized. In the study for calculating sample size, the following equation was adopted (Gogtay, Indian J Ophthalmol: 2010;58:517-518)

$$n = [(Z\alpha + Z\beta)^2 \times \sigma^2] / d^2$$

where n is the calculated sample size, Z_α equals 1.96 for confidence level of 95%, Z_β equals 0.84 at 80% power of study, σ is the standard deviation of the population being studied, and d is the mean difference in the effect that to be clinically detected. Based on the aforementioned equation, a total of 42 patients, divided as 21 patients in each treatment group, underwent the study, considering 10% rate of attrition. The primary outcome was fairness in the clinical cure rate, and, for the secondary outcomes, the fungal burden reduction rate was considered to be >50% of the pre-treatment value, the combined clinical and microbiological cure rate, and the acceptability of the products in the trial. The significance level considered was $P<0.05$%

Selection Criteria

The participants, aged 48 to 71 years, with denture stomatitis fulfilling Newton's criteria were recruited from the outpatient department of Prosthodontics. The participants were enrolled at the JSS Dental College and Hospital, between January and June 2020 and were registered at Clinical trials.gov REF/2020/07/035325. The volunteers agreeing to participate in the study, signed the informed consent form based on inclusion and exclusion criteria (Rs et al., 2015). Inclusion Criteria include (1) patients rehabilitated with removable complete dentures for more than 1 year; (2) patients willing to participate in the study; (3) patients with denture stomatitis; (4) patients who have not used anti-fungal drugs and finally (5) patients who are not suffering from any systemic disease. Exclusion criteria include (1) patients unwilling to participate in the study; (2) patients already on antibiotics or antifungal therapy; (3) patients suffering from other dental conditions, systemic diseases, or with an immune-compromised condition; (4) volunteers under medications initiated less than 3 months before registration and finally (5) volunteers who participated in other clinical studies or have taken a trial drug within the last one month of screening.

Baseline data on years of edentulism, denture age, oral/denture hygiene, and nocturnal wear was assessed with a reliable validated self-administrated questionnaire. Evaluation of vertical dimension of occlusion, stability, retention of the maxillary prosthesis, and the resiliency of the maxillary edentulous ridge, was carried out according to standard prosthodontic criteria. A clinical intraoral examination was conducted for the diagnosis of denture stomatitis. The hard palate was examined at the initial visit for the presence of erythematous spots or areas, papillary hyperplasia. The mucosal lesions were classified as Newton-type I (localized petechiae); Type II (more diffuse erythema involving part of the area covered by the prosthesis); and Type III (erythema with papillary hyperplasia in the area covered by the prosthesis) (Figure S1). Photographs of the palatal mucosa were captured with a Nikon D90 camera (105 mm f/2.8 D, macro flash SB-21; Nikon Co., Tokyo, Japan). These snapshots were employed to acquire a diagnostic agreement from research team members.

Randomization

Randomization was done by computer-generated random numbers. The group allocation also included volunteer number and day of swab collection.

Table S1. Drug interactions of clove and cinnamon extracts against *C. albicans in vitro.*

Clove (ug/mL)	Cinnamon (ug/mL)	FIC_{clove}	$FIC_{cinnamon}$	FICI	Inerpretation
512	64	1.00	1.00	2.00	Antagonist
256	32	0.50	0.50	1.00	Additive
128	8	0.25	0.13	0.38	Synergistic
64	16	0.13	0.25	0.38	Synergistic
32	4	0.06	0.06	0.13	Synergistic

Table S2. Physical appearance of Emulgel

Formulation Code	Properties			
	Appearance	Homogeneity	Grittiness	Smoothness
F1	White	Good	Nil	Good
F2	White	Good	Nil	Good
F3	White	Good	Nil	Good
F4	White	Good	Nil	Good
F5	White	Good	Nil	Good
F6	White	Good	Nil	Good
F7	White	Good	Nil	Good
F8	White	Good	Nil	Good
F9	White	Good	Nil	Good

Table S3. Stability data of clove/cinnamon extracts-loaded emulgel at 25 ± 2˚C and 40 ± 2°C

Parameter	Fresh F2	Stability at 25 ± 2˚C		Stability at 40 ± 2°C	
		1 month	3 months	1 month	3 months
Physical appearance	White, homogenous	White, homogenous	White, homogenous	White, homogenous	White, homogenous
pH	6.26 ± 0.06	6.21 ± 0.05	6.15 ± 0.09	6.12 ± 0.11	6.05 ± 0.10
Drug content (Clove)	97.9 ± 0.09%	97.1 ± 0.7%	96.2 ± 1.5%	95.8 ± 1.7%	94.3 ± 2.5%
Drug content (Cinnamon)	96.4 ± 0.24%	96.1 ± 0.41%	95.3 ± 0.76%	93.8 ± 2.5%	92.9 ± 3.1%

Table S4. Chi-Square tests of Clinical response to treatment between both the groups

**Group**	**Chi-Square test**	**Value**	**Df**	***P* value**
Test	Pearson Chi-Square	33.127	9	0.001
Control	Pearson Chi-Square	27.333	9	0.001
Total	Pearson Chi-Square	51.886	9	0.001

*P <0.05, **P <0.01 and *** P <0.001

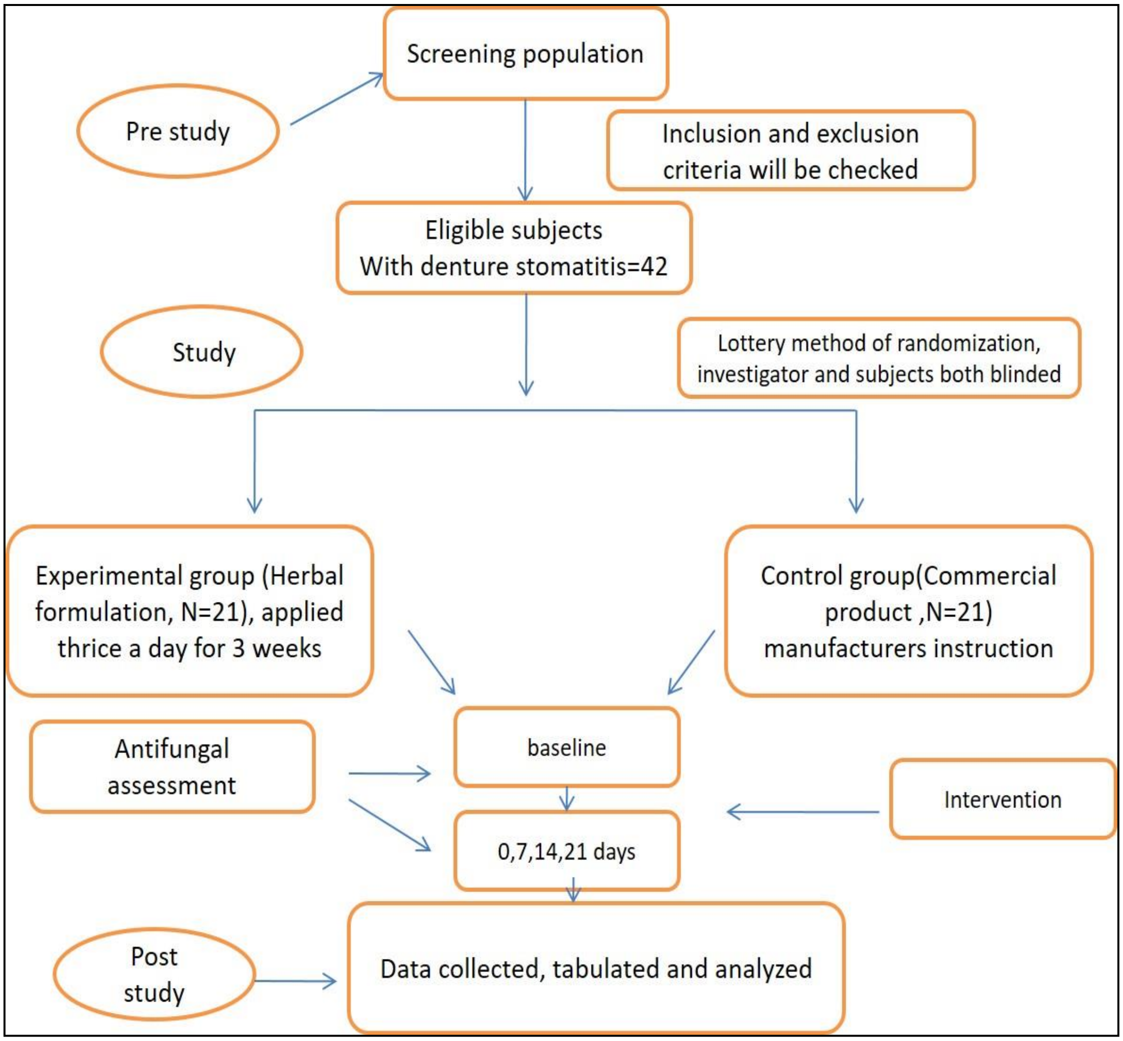

Figure S1. CONSORT CHART

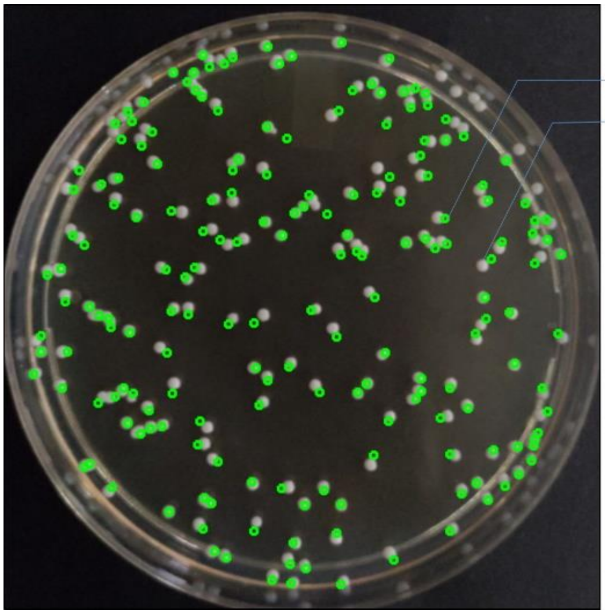

Figure S2. Colony Counting on SDA by using Colony Count Software.
